# Electroplated waveguides to enhance DNP and EPR spectra of silicon and diamond particles

**DOI:** 10.5194/mr-3-203-2022

**Published:** 2022-10-06

**Authors:** Aaron Himmler, Mohammed M. Albannay, Gevin von Witte, Sebastian Kozerke, Matthias Ernst

**Affiliations:** 1 ETH Zurich, Laboratory of Physical Chemistry, Zurich 8093, Switzerland; 2 University and ETH Zurich, Institute for Biomedical Engineering, Zurich 8092, Switzerland

## Abstract

Electroplating the waveguide of a 7 T polarizer in a simple innovative way increased microwave power delivered to the sample by 3.1 dB. Silicon particles, while interesting for hyperpolarized MRI applications, are challenging to polarize due to inefficient microwave multipliers at the electron Larmor frequency at high magnetic fields and fast electronic relaxation times. Improving microwave transmission directly translates to more efficient EPR excitation at high-field, low-temperature conditions and promises faster and higher ${}^{29}$ Si polarization buildup through dynamic nuclear polarization (DNP).

## 1 Introduction

Nuclear magnetic resonance (NMR) and electron paramagnetic resonance (EPR) spectroscopy are two well-established methods for studying the structure of materials such as silicon-based micro- and nanoparticles (Ha et al., 2019; Hahn et al., 2008; Tasciotti et al., 2008). Direct ${}^{29}$ Si NMR detection is challenging due to the low thermal polarization, low natural abundance of 4.7 %, and long nuclear longitudinal relaxation times of ${}^{29}$ Si (Dementyev et al., 2008). Dynamic nuclear polarization (DNP) at low temperatures ( <4 K) and high static magnetic fields significantly enhances sensitivity but requires long buildup times for the polarization (Kwiatkowski et al., 2018b, 2017; Cassidy et al., 2013b), causing a low experimental repetition rate. Nevertheless, hyperpolarized particles offer great potential for diagnostic use in in vivo magnetic resonance imaging (MRI) (Luu et al., 2020; Cassidy et al., 2013a; Kwiatkowski et al., 2017; Whiting et al., 2015; Aptekar et al., 2009; Atkins et al., 2013). Accordingly, development of polarization methods and particle optimization has been an important field of research (Ha et al., 2019; Kwiatkowski et al., 2018b; Dementyev et al., 2008; Cassidy et al., 2013b). In contrast to exogenous radicals traditionally used in DNP methods (Jähnig et al., 2019, 2017), the DNP process in silicon particles is driven by endogenous free radicals concentrated in the outer particle surface at the interface between SiO ${}_{2}$ and Si in the form of dangling bonds (Dementyev et al., 2008; Ha et al., 2019; Cassidy et al., 2013b). These comparably fast-relaxing electrons require intense microwave irradiation to sufficiently saturate the electron transitions. Therefore, low microwave intensities only excite a small fraction of the electron transitions at any given frequency, which limits the full potential of EPR spectroscopy, as well as continuous wave or pulsed applications for DNP (Tan et al., 2019). Solid-state microwave sources are inefficient at increasingly higher EPR frequencies, leading to limited microwave intensities in the sample space. Gyrotrons are established systems to generate high microwave intensities (Blank et al., 2020) commonly used in magic-angle spinning (MAS) DNP systems but due to their much higher cost are not a viable solution for dissolution DNP. Transmission of the microwaves to the sample space can be achieved by waveguides or corrugated waveguides with low losses (Nanni et al., 2012; de Rijk et al., 2011) or using quasi-optic transmission of the microwaves (Siaw et al., 2016). The desire towards implementing corrugated waveguides in low-temperature DNP setups (de Rijk, 2013; Leggett et al., 2010) shows that minimizing transmission losses is an important strategy to maximize microwave power in the sample.

Classically, microwaves are delivered to the DNP sample at cryogenic temperatures by a stainless-steel waveguide, to minimize heat conduction to the sample space. Ohmic losses associated with stainless steel at the microwave frequency result in significant transmission losses. The magnet bore length typically dictates the waveguide length needed to couple the microwave source output to the sample. The ideal waveguide minimizes transmission losses while at the same time maximizing thermal isolation between the room-temperature microwave source and the liquid-helium-cooled sample space. The waveguide conductivity and waveguide aperture directly dictate the observed transmission losses and propagation mode (Zinke, 1990). Under the right circumstances, corrugated waveguides offer an elegant solution to reduce conductive losses (Leggett et al., 2010; de Rijk, 2013; Nanni et al., 2012; de Rijk et al., 2011). Mode converters can also be utilized to exploit propagation modes that experience less attenuation through certain waveguide geometries (Yu and Chang, 2005). Electroplating the inside of the waveguide is another common solution to reduce transmission losses and subsequently improve EPR sensitivity (Albannay et al., 2019; Li, 2020). In our current DNP polarizer setup (Jähnig, 2018), we found electroplating to be the simplest solution, due to ease of adoption into the existing probe design (Jähnig et al., 2017). In this work a simple innovative method is demonstrated for electroplating waveguides on a lab bench. By exploiting the skin depth effect, a thin silver layer plated inside a stainless-steel waveguide can reduce transmission losses whilst preserving thermal isolation (Petencin, 2015), thereby facilitating higher microwave intensities at the sample space.

## 2 Materials and methods

### 2.1 Instrumentation

Experiments were performed on a home-built solid-state DNP polarizer (Jähnig, 2018). The polarizer uses a wide-bore NMR magnet (Bruker BioSpin AG, Switzerland) charged to 7 T corresponding to an EPR frequency of 197 GHz. The solid-state microwave source is based on a frequency synthesizer fed into an amplifier–multiplier chain (197 GHz Tx; Virginia Diodes Inc, VA, USA) resulting in a frequency range of 197±5 GHz and a power output up to 200 mW at the source. A NI-USB 6525 data acquisition device DAQ (National Instruments, TX, USA) controls the microwave power attenuation, while the USB-controlled synthesizer allows for frequency modulation schemes. The magnet houses a Spectrostat cryostat (Oxford Instruments, Oxford, UK) capable of reaching a base temperature of 3.3 K in continuous helium-flow mode.

The DNP probe (Jähnig, 2018) is 106 cm long and constructed from a fiberglass tube (OD =20 mm, ID =18 mm) terminated into an aluminum-machined top plate featuring three feed-through connectors (LEMO, Ecublens, Switzerland) for sensors and longitudinal detection (LOD) of EPR. The top flange is screwed onto the cryostat and sealed with an O-ring. Helium-level sensing is capacitively performed using two concentric thin-walled brass tubes ( L=220 mm, OD1 =9 mm, OD2 =3 mm) affixed at the bottom of the DNP probe. The microwave source couples to a WR-05 to WR-28 taper at the top flange. Subsequently, two overmoded WR-28 waveguides couple microwaves to the sample space. The first waveguide section, 39.2 cm long, is made from copper, while the lower waveguide, 65.5 cm long, is made from 304 stainless steel to limit heat transfer to the sample space. Only the latter part of the waveguide was electroplated. A WR-28 to 1.5 mm circular taper subsequently couples the waveguide to a 90 ${}^{\circ }$ bend and a horn antenna to improve sample irradiation. The fiberglass tube concentrically accommodates an NMR sample insert and seals the top plate with two O-rings. The insert is primarily constructed from a semi-rigid coaxial cable featuring a nine-turn solenoid coil (ID =4 mm) that hosts a 75 µL PTFE sample cup. The coil is accessed via an N-type connector atop the coaxial cable.

**Figure 1:**
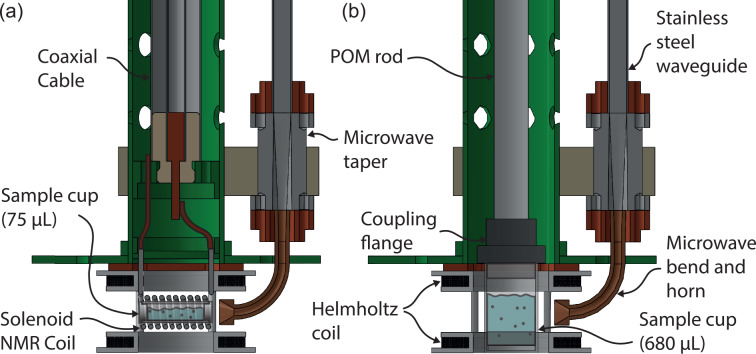
Slice through the bottom of the 7 T DNP probe in DNP-NMR configuration **(a)** and EPR configuration **(b)**. The split Helmholtz coil is wound inside of two U-profiled polychlorotrifluoroethylene discs. These accommodate enough space to insert the NMR coil as well as a 680 µL sample cup.

To facilitate EPR detection, a split Helmholtz coil (1500 turns) was wound using 0.1 mm copper wire and mounted at the bottom of the DNP probe (Fig. 1). The coils are connected in series and accessed via the top flange. For this purpose, an EPR insert replaces the NMR insert. It is constructed from a polyoxymethylene (POM) rod which terminates in a sample cup coupling flange. The sample cup is expanded to 680 µL and friction-fits into the coupling flange. Like the NMR sample insert, the EPR sample insert seals the probe's top plate with two O-rings.

### 2.2 Bench characterization

Transmission losses at 197 GHz were characterized on the bench using the 197 GHz DNP transmitter system described above. The microwave source couples to a WR-05 to WR-28 taper via a WR-5 isolator. For power measurements, a second taper couples the waveguide to a diode detector (WR5.1ZBDF, Virginia Diodes Inc, VA, USA). Transmission losses are measured by inserting the WR-28 steel waveguide, 65.5 cm long, between both tapers, before and after plating.

As an additional validation, point transmission losses were measured at 94 GHz, which corresponds to the microwave frequency used in our 3.35 T polarizer. Here the transmitter system (Virginia Diodes Inc, VA, USA) is coupled to a pair of WR-10/WR-28 tapers, which terminate in a diode detector (ZBDA-10/94/20, ELVA-1 Millimeter Wave Division, St. Petersburg, Russia). Losses are measured by inserting a 34 cm WR-28 steel waveguide between both tapers, before and after plating. Both microwave sources are operated at maximum output power, while both detectors are protected by appropriate attenuators.

**Figure 2:**
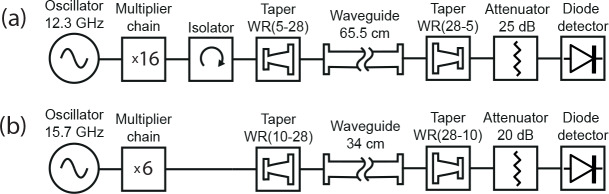
Schematic setup for measuring transmission losses at **(a)** 197 GHz and **(b)** 94 GHz microwave frequency.

### 2.3 Electroplating

**Figure 3:**
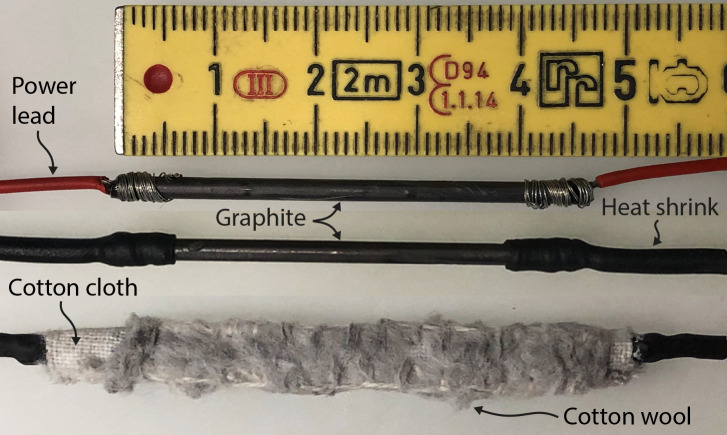
Three steps of constructing the plating tool based on pencil graphite as described above. In the bottom, a thin layer of cotton wool is additionally sewn to the fabric for the solution to easily also reach the waveguides' inner corners.

To ensure an even coat via electroplating, the inner surface of the waveguides was first abrasively polished with fine steel-wool then degreased with a water-based degreaser (Electro cleaner, Betzmann Galvanik, Pfullendorf, Germany) and rinsed with distilled water. No surface etching was required. The plating tool (Fig. 3) is constructed using a conductor wrapped in cloth, which is soaked with the plating solutions. The conductor is a graphite rod ( 2.15×1.55 mm ${}^{2}$ ) that fits in a WR-28 waveguide while accommodating a layer of cloth material. Both ends of the graphite rod are then connected to power leads and mechanically secured using heat shrink and glue. One of the leads is used to conduct current, while both are necessary to pull the tool through the waveguide. Finally, cotton cloth is wrapped around the exposed graphite section and sewn in place, thereby filling the waveguide aperture. One plating tool was built for each of the two solutions (10 g L ${}^{-1}$ gold, 25 g L ${}^{-1}$ silver). These solutions (Betzmann Galvanik, Pfullendorf, Germany) are optimized for pen galvanization and are based on aqueous solutions of silver nitrate or potassium dicyanoaurate, respectively. The waveguide is then connected to the negative pole of a DC power supply (Dr.K.Witmer, TF-72/1), while the positive pole is connected to one of the power leads of the plating tool. The tool is impregnated with the selected solution and inserted in the waveguide while a constant voltage is applied. After the plating process starts, the plating tool is pulled slowly (about 3 cm s ${}^{-1}$ ) through the waveguide to create an even metal layer. After every fifth pass, a few drops of fresh solution are added on the cloth. First, a thin gold layer is applied as a base coat with 100 passes using 5.5 V. After rinsing with distilled water, the conductive coat of silver is added with 300 passes at 3 V. This whole process is performed on a lab-bench at room temperature, without the need for elaborate equipment or large submersion tanks. To verify sufficient silver coat thickness, waveguide attenuation is measured at regular intervals, as described above.

### 2.4 LOD-EPR

**Figure 4:**
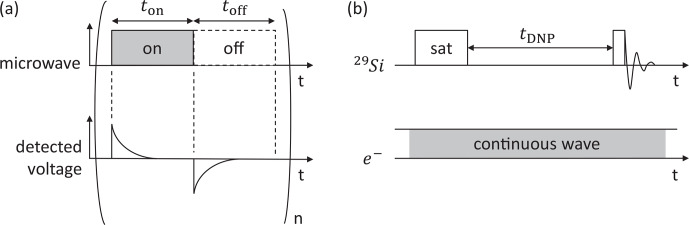
**(a)** Schematic representation of LOD-EPR experiments. The microwave is modulated on and off n times, while the detected voltage from the LOD coil is averaged for every cycle. **(b)** Pulse sequence for DNP-enhanced NMR experiments. Both sequences are repeated for different microwave frequencies to obtain the EPR spectrum and the DNP profile respectively.

Silicon powder (P28A002, 1–20 µm , Alfa Aesar, characterized by Kwiatkowski et al. (2018b) and diamonds (MSY 8–12 µm , Microdiamant AG, characterized by Kwiatkowski et al. (2018a) were packed (1.25 and 2.13 g mL ${}^{-1}$ ) in 680 µL cylindrical sample cups and placed in the EPR insert, one at a time, for the experiments. The cryostat was cooled to 20 K. The microwave output was gated in a rectangular pattern (Fig. 4) using a DAQ with a 50 % duty cycle ( $t_{\mathrm{on}}=t_{\mathrm{off}}=500$ µs ). In every cycle, the induced voltage signal was amplified (see Jähnig et al., 2019) and sampled at 1 MHz by the DAQ. To improve the signal-to-noise ratio (SNR), n=2000 measurement cycles were averaged. Subsequently, after a 2 s delay, the microwave frequency was changed to reacquire another point of the EPR spectrum. Relaxation constants were obtained by fitting the raw data with an exponential decay. To construct the EPR spectrum, the induced voltage integral was calculated for each frequency. Power-dependent experiments were performed in the same manner as a series of experiments but restricted to the center frequency of the respective EPR lines. For every LOD experiment, the microwave power was automatically set at the amplifier–multiplier chain (AMC) by a constant voltage from the DAQ.

### 2.5 DNP-NMR

Silicon powder (P28A002, 1–20 µm , Alfa Aesar) was packed in a 75 µL sample cup and loaded in the coil of the NMR insert. The cryostat was cooled to 3.4 K. The sample was continuously irradiated using the maximal source power, while the output frequency was set automatically at the beginning of each NMR experiment. All NMR measurements were performed using a Bruker Avance III HD console using a center frequency of 59.43 MHz for ${}^{29}$ Si. The pulse sequence consists of 32 saturation pulses (200 W, 6.5 µs pulse length, 3.45 ms delay) followed by a buildup time ( $t_{\mathrm{DNP}}=300$ s) and a π/2 detection pulse (200 W, 6.5 µs ). Each free-induction decay (FID) was recorded with 2048 points and a dwell time of 2 µs . This experiment was repeated for each microwave frequency. The DNP intensity was calculated using the integral over the NMR line.

## 3 Results and discussion

**Figure 5:**
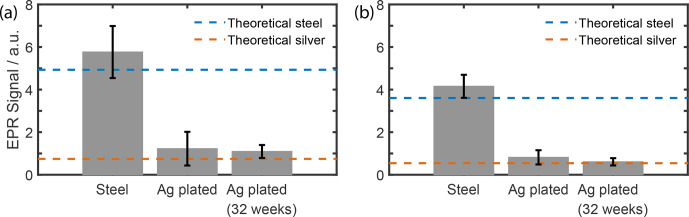
Microwave attenuation before and after plating of **(a)** 65.5 cm steel waveguide at 197 GHz and **(b)** 34 cm steel waveguide at 94 GHz. The dotted lines indicate the theoretical attenuation (Zinke, 1990) of a WR28 waveguide made from pure steel or pure silver.

WR28 stainless-steel waveguides were internally electroplated with silver to reduce transmission losses and verified at 94 and 197 GHz. Benchtop measurements show that plating reduces the losses by 3.6 and 4.7 dB m ${}^{-1}$ at 94 and 197 GHz, respectively (Fig. 5). Given the waveguide section, 655 mm long, used in our 7 T DNP probe, plating reduced losses by 3.1 dB. The minimum attenuation of the entire microwave chain at room temperature is now 3.7 dB. At liquid-helium temperatures, the electrical resistance is lower (Matula, 1979); thus the attenuation will be reduced in the colder parts of the waveguide. The skin depth of the microwave will also shrink with lower resistance (Zinke, 1990). Therefore, a thinner silver layer might give the same results. Testing the waveguide 8 months later shows no significant change in attenuation, namely 1.2 to 1.1 dB m ${}^{-1}$ at 197 GHz and 0.8 to 0.6 dB m ${}^{-1}$ at 94 GHz. The consistently low numbers indicate good plate adhesion during electroplating and low corrosion. No change in temperature or helium consumption was observed during experiments.

The presented waveguide plating method is easy and cost-effective to reduce transmission losses and enhance the performance of low-temperature EPR and DNP setups. This method also works with curved, nonsymmetric, and long waveguides ( >50 cm), where classical electroplating can be difficult. The observed changes in attenuation are similar to what is expected by theoretical calculations (Zinke, 1990). Following this prediction, the increased conductivity after silver plating offers higher benefits at higher frequencies.

**Figure 6:**
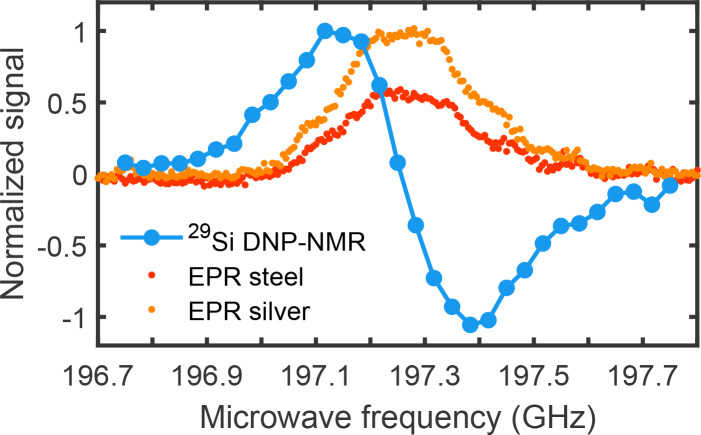
LOD-EPR spectra of silicon particles (AA1-20 µm ), with microwave power according to the steel waveguide (red) and silver-plated waveguide (orange) at 20 K. The corresponding ${}^{29}$ Si DNP profile (blue) is measured at 3.4 K with a buildup time of 300 s to obtain sufficient signal.

**Figure 7:**
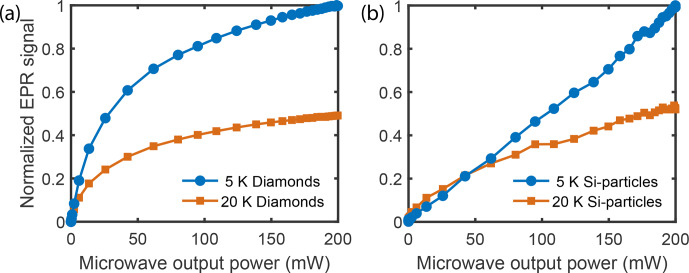
Power-dependent LOD-EPR signal at 5 and 20 K using the plated waveguide. **(a)** Diamond particles. **(b)** Silicon particles.

Silver-plating the waveguide improved LOD-EPR sensitivity, when measuring silicon particles. According to the EPR spectra in Fig. 6, the EPR signal increases by 74 % using the same microwave power settings. The EPR lines agree well with the ${}^{29}$ Si DNP profile. The power dependence of EPR signals can be measured by a saturation curve, as seen in Fig. 7. Here it is possible to assess how increasing microwave power changes the signal. The EPR saturation curves were measured for silicon and diamond particles at their respective center frequencies. The EPR signal of silicon shows an unexpected near-linear signal increase up to 200 mW power, possibly linked to the fast electronic relaxation times that require higher microwave power for complete saturation (Yoon et al., 2019; Rupp et al., 1978; Lund et al., 2009). The strong power dependence has already been shown in the low-temperature 3.35 T DNP setup, where the maximum achievable ${}^{29}$ Si signal increases strongly for input powers beyond 200 mW (Kwiatkowski et al., 2017). The signal increase through the waveguide plating is much less pronounced in diamond, with a below-linear power dependence, within the available power limits. The discrepancy between the two samples is more pronounced at lower temperatures.

Longitudinal electron relaxation times were obtained by fitting the time-resolved LOD data with an exponential decay (Table S1 in the Supplement). Here the decay rates at the center frequency of the respective EPR lines are discussed for a temperature of 20 K. While a solution of 50 mM 4-oxo-TEMPO in 1:1 ( v/v ) water / glycerin shows an electron $T_{1}$ decay of around 520 µs , micro diamonds and silicon particles show a bi-exponential decay with two different time constants. The slow components (462 and 187 µs respectively) form the majority of the EPR signal. The fast component (about 10 µs ) can be detected at all EPR frequencies over the whole accessible microwave range as a small background EPR signal. We speculate that this component might come from closely clustered defect electrons on the surface that result in an extremely broad line. The observed electron relaxation time in LOD experiments, after (partial) saturation of the EPR line, might be faster than the real electron relaxation time. This can be the consequence of spectral spin diffusion of magnetization from neighboring, not saturated parts of the line, which adds to the recovery of the magnetization towards the thermal equilibrium and makes the relaxation appear faster than it is. Nonetheless, the faster electron $T_{1}$ time in silicon, compared to diamond, does suggest a higher required power to saturate the lines (Yoon et al., 2019), as seen in Fig. 7.

In LOD-EPR experiments the signal intensity of silicon particles is only a fraction of the signal intensity that can be detected from diamonds or TEMPO (Fig. S1 in the Supplement), both of which are known for their high DNP signal enhancement (Jähnig et al., 2019; Kwiatkowski et al., 2018a). For silicon particles the lower signal intensity can be explained by the lower number of defects (Kwiatkowski et al., 2018b) (about 10 mM in our sample) and by the fact that the microwave power is too low to excite all spins. Higher microwave power would increase the saturation, but increased continuous-wave (CW) irradiation can also raise the sample temperature (Hope et al., 2021), resulting in lower Boltzmann polarization and ultimately lower signal intensity.

## 4 Conclusion

We have shown a simple method to silver-plate waveguides in order to reduce the transmission losses. This method can be applied on a lab bench without special equipment. The increased microwave power thereby delivered to the sample has been demonstrated to be beneficial for experiments using fast-relaxing radicals and potentially also for pulsed experiments. Using the increased microwave power in the sample space and the expanded sample cup allowed us to detect the EPR spectrum of silicon particles. Due to the lower number of defects and shorter relaxation times, this was previously not possible with our experimental setup. The almost-linear power-dependent increase in the silicon LOD signal intensity suggests that even more microwave power is necessary to saturate all spins.

## Supplement

10.5194/mr-3-203-2022-supplement The supplement related to this article is available online at: https://doi.org/10.5194/mr-3-203-2022-supplement.

## Data Availability

Experimental data are available from the corresponding author on request.
