# Supplementary material for: Electroplated waveguides to enhance DNP and EPR spectra of silicon and diamond particles
Source: Magn Reson (Gott). 2022 Oct 6;3(2):203–9. doi: 10.5194/mr-3-203-2022 (PMC10539804; doi:10.5194/mr-3-203-2022)
Supplement: The supplement related to this article is available online at: https://doi.org/10.5194/mr-3-203-2022-supplement. [file mr-3-203-supplement.pdf]

Supplement of Magn. Reson., 3, 203–209, 2022
https://doi.org/10.5194/mr-3-203-2022-supplement
© Author(s) 2022. CC BY 4.0 License.

Open Access
MAGNETIC RESONANCE

*Supplement of*

# Electroplated waveguides to enhance DNP and EPR spectra of silicon and diamond particles

**Aaron Himmler et al.**

*Correspondence to:* Matthias Ernst (maer@ethz.ch)

The copyright of individual parts of the supplement might differ from the article licence.

Table S1: Experimental $T_{1e}$ relaxation times of different radicals at different temperatures, measured by LOD, 7 T. Samples: 50 mM 4-oxo-TEMP in 1:1 (*v*/*v*) water/glycerin, nano-diamonds (636428-1G, <10 nm, Aldrich), micro-diamonds (MSY 8-12 µm, Microdiamant AG), silicon particles (P28A002, 1-20 µm, Alfa Aesar).

| **Temperature** | $T_{1e}$ **TEMPO** | $T_{1e}$ **nano-diamonds** | $T_{1e}$ **micro-diamonds** | $T_{1e}$ **silicon particles** |
|---|---|---|---|---|
| 5 K | 605.4 µs | 77.0 µs | 490.7 µs (99.3%)<br>17.0 µs (0.7%) | 359.5 µs (99.6%)<br>8.8 µs (0.4%) |
| 7 K | 599.3 µs | 55.4 µs | 496.4 µs (99.4%)<br>14.2 µs (0.6%) | 297.9 µs (96.1%)<br>15.9 µs (3.9%) |
| 10 K | 591.3 µs | 37.5 µs | 485.5 µs (98.9%)<br>24.5 µs (1.1%) | 242.4 µs (87.0%)<br>19.6 µs (13.0%) |
| 15 K | 566.6 µs | 27.3 µs | 459.5 µs (99.1%)<br>17.7 µs (0.9%) | 209.8 µs (90.6%)<br>12.6 µs (9.4%) |
| 20 K | 518.4 µs | 22.4 µs | 462.2 µs (99.5%)<br>11.6 µs (0.5%) | 186.9 µs (90.6%)<br>9.4 µs (9.4%) |

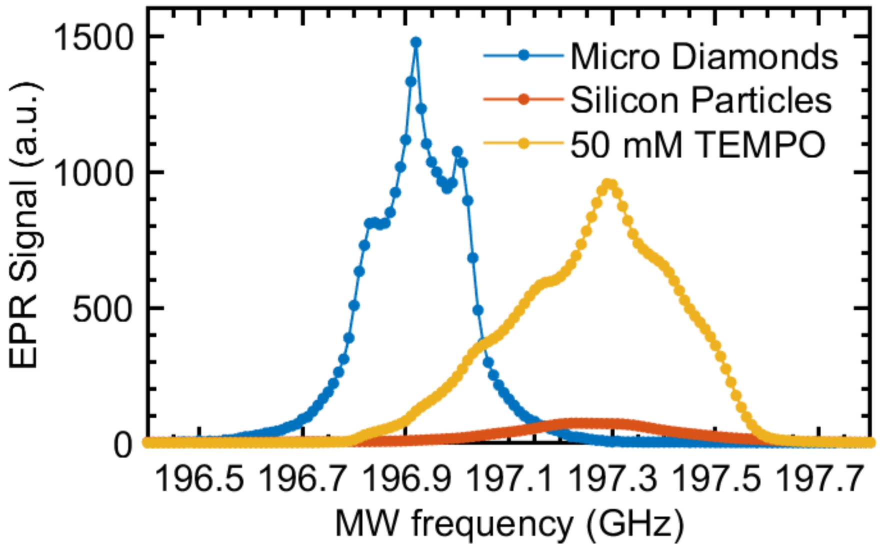


Fig. S1: LOD-EPR spectra of micro diamonds (blue), silicon particles (red) and 50 mM 4-oxo-TEMPO in 1:1 (*v*/*v*) water/glycerin (yellow), 7 T at 20 K, 200 mW microwave output power.

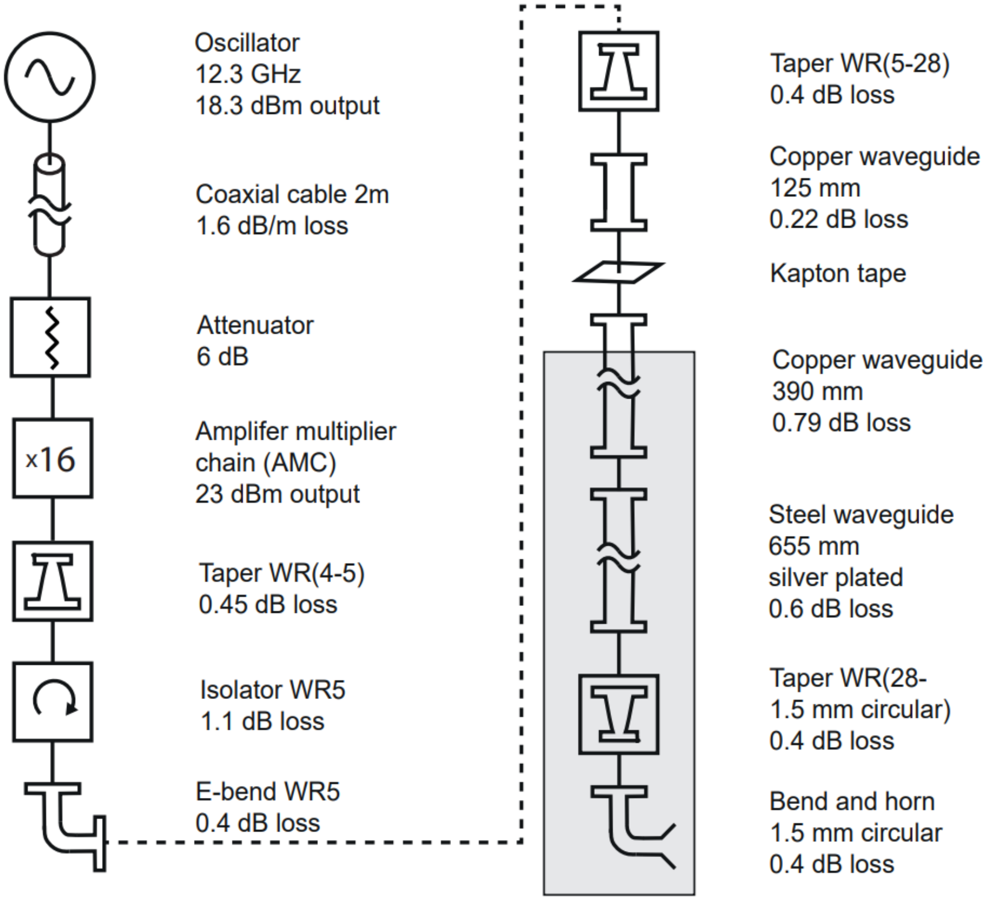


Fig. S2: Schematic depiction of the microwave chain with all components of the 7 T DNP polarizer. Components in the grey box are located inside the cryostat, which in turn sits in the 7 T magnet.
